# Association of Postoperative Delirium and Perioperative Factors With Frailty in Laparoscopic Gastrointestinal Surgery: A Retrospective Observational Study

**DOI:** 10.7759/cureus.94914

**Published:** 2025-10-19

**Authors:** Naoko Tachibana, Yuta Mitobe, Fukuyo Taichi

**Affiliations:** 1 Nursing, Toho University Omori Medical Center, Tokyo, JPN; 2 Perianesthesia Nursing, International University of Health and Welfare Graduate School, Tokyo, JPN

**Keywords:** asa-ps, delirium, frailty, gastrointestinal surgical patients, pain

## Abstract

Background: In the current era of advancing population aging, perioperative management of elderly patients has become a crucial challenge. Frailty is recognized as a condition associated with poor postoperative outcomes, and, particularly, postoperative delirium is an urgent issue for healthcare settings as a major complication in elderly patients. Currently, there is no established international standard for assessing frailty; thus, further understanding in this field is required.

Objective: This study aimed to elucidate the association between preoperative frailty and the incidence of postoperative delirium and pain in patients undergoing gastrointestinal laparoscopic surgery.

Methods: Patients aged 70 years and older who underwent gastrointestinal laparoscopic surgery between July 2019 and May 2022 were assessed using the clinical frailty scale (CFS). Based on the CFS score, participants were categorized into two groups: non-frail (CFS≤3) and frail (CFS≥4). Statistical analyses were conducted to compare outcomes between the groups.

Results: A total of 323 cases were analyzed, comprising 228 patients in the non-frail group and 95 in the frail group. Logistic regression analysis was performed for variables that showed significant differences. In univariate analysis, significant differences were found in six items: age, length of hospital stay, ASA Physical Status Classification (ASA-PS), presence of physical restraint, postoperative delirium screening tool (DST), and postoperative pain. Multivariate analysis confirmed that all six variables remained significantly associated.

Conclusion: Among frail patients undergoing gastrointestinal laparoscopic surgery, key perioperative factors associated with frailty included advanced age, prolonged hospitalization, higher ASA-PS scores, use of physical restraints, increased DST positivity rates postoperatively, and greater postoperative pain. Each of these factors is a potential contributor to delirium, suggesting that frail individuals are at heightened risk of developing delirium due to these associated variables.

## Introduction

The proportion of individuals aged 65 and older in Japan continues to rise, and the aging rate is projected to increase further in the coming years. The prevalence of frailty among community-dwelling older adults ranges from 1.5% to 17.9%, and it has been identified as a prognostic factor associated with extended postoperative hospitalization, increased incidence of complications, and elevated mortality rates. Several perioperative factors, such as postoperative pain and the use of physical restraints, have been shown to be associated with the risk of delirium onset. These are considered potential targets for preventive interventions, as 30-40% of delirium cases are thought to be preventable [1]. For example, effective pain management and the avoidance of physical restraints may contribute to the prevention of delirium. Despite its importance, an internationally established standard for frailty assessment still does not exist. Globally, diverse assessment tools are in use, such as the Gerontopole Frailty Screening Tool (GFST) and the Basic Checklist [2]. In Japan, multiple assessment methods, including the J-CHS (Japanese version of the Cardiovascular Health Study) criteria adapted by the Ministry of Health, Labour and Welfare, are also employed [3]. In the present study, given its retrospective nature, the Clinical Frailty Scale (CFS) was adopted because it does not require detailed information and is simple to evaluate [4].

## Materials and methods

Study design

This study was conducted as a single-center, retrospective observational study.

Study population

**Figure 1:**
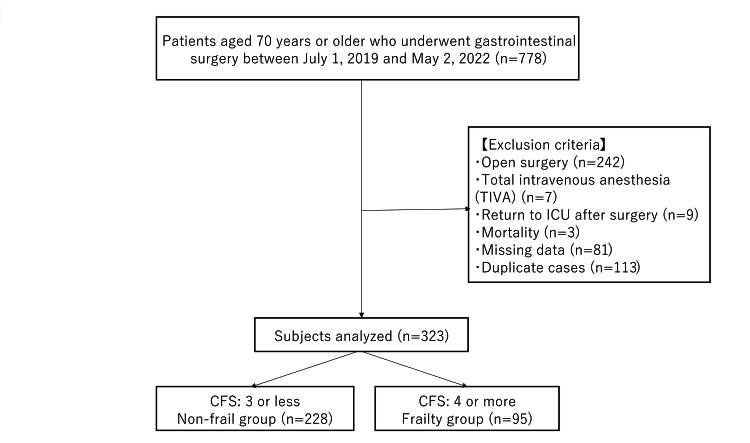
Flowchart of the study population analysis CFS: Clinical Frailty Scale

The study population consisted of patients aged 70 years and older who underwent gastrointestinal surgery at the Toho University Omori Medical Center between July 1, 2019, and May 2, 2022. Among 778 patients aged 70 years or older who underwent gastrointestinal surgery, 323 patients who received laparoscopic procedures were included in the analysis. Based on the CFS score, participants were categorized into two groups: non-frail (CFS≤3) and frail (CFS≥4). Statistical analyses were conducted to compare outcomes between the groups. The CFS cutoff of ≤3 versus ≥4 was chosen based on prior validation studies. Specifically, CFS ≥4 is often considered to represent a "vulnerable" state, associated with an increased risk of future frailty progression, falls, and hospital admissions [5]. The exclusion criteria comprised 242 patients who underwent open surgery, seven patients who received total intravenous anesthesia (TIVA), nine patients who were admitted to the Intensive Care Unit (ICU) postoperatively, three cases of mortality, and 81 cases with missing data, including those who underwent exploratory laparotomy due to inoperable conditions. In the ICU, the Confusion Assessment Method for the ICU (CAM-ICU) is used for delirium assessment. Since different assessment tools are used in the general wards (which use the delirium screening tool, DST) and the ICU, ICU patients were excluded. The reason for excluding TIVA cases was that, according to a study by Miller et al., there was no difference in the incidence of postoperative cognitive dysfunction (POCD) between inhalation anesthesia and TIVA. However, that study concluded that the evidence level was very low due to issues such as bias and differing measurement points across studies [6]. Since the number of TIVA cases was also small, we opted for inhalation anesthesia only to avoid bias. To eliminate duplication, 113 patients with overlapping surgical records were excluded, ensuring that each patient was counted only once (Figure 1).

Ethical considerations

This study was conducted in accordance with the ethical principles outlined in the “Ethical Guidelines for Medical and Health Research Involving Human Subjects.” Ethical approval was obtained from the Ethics Committee of Toho University Omori Medical Center (Approval No.: M23187). In addition, an “Information Disclosure Document for Clinical Research” outlining participants’ right to withdraw from the study at any time was published on the website of Toho University Omori Medical Center to ensure ethical transparency.

Assessment items

Preoperative Assessment Variables

Data collected for preoperative evaluation included age, sex, American Society of Anesthesiologists Physical Status (ASA-PS), height, weight, body mass index (BMI), medication history, smoking history, and pre-existing comorbidities (hypertension, diabetes mellitus, ischemic heart disease, cerebrovascular disease, dementia, psychiatric disorders, and alcohol dependence). Additionally, the presence or absence of a positive screening result on the DST at admission was recorded. Laboratory data included C-reactive protein (CRP), total protein, albumin, blood urea nitrogen, creatinine, estimated glomerular filtration rate (eGFR), white blood cell count, red blood cell count, hemoglobin, hematocrit, and platelet count.

DST Screening

The DST is a subjective nurse-administered screening instrument for the detection of delirium [7]. Item A focused on “level of awareness, arousal, and cognition,” and seven items were evaluated: sense of reality, decreased activity, excitement, mood swings, disruption of sleep-wake rhythm, hallucinations, and delusions. Item B, “Cognitive Changes,” assessed two items: disorientation and memory impairment. Item C focused on “symptom variability” and evaluated two items: pattern of onset and intraday symptom variability. If at least one of A, B, and C was applicable, the patient was evaluated as possibly delirious.

Intraoperative Assessment Variables

Data collected during the intraoperative period included whether the procedure was elective or emergent, duration of anesthesia, duration of surgery, presence or absence of epidural catheter placement, dosages of administered agents (sevoflurane, propofol, fentanyl, and remifentanil), estimated blood loss, and whether blood transfusions were administered.

Postoperative Assessment Variables

Postoperative variables included length of hospital stay, time to bed release, time to initiation of ambulation, postoperative pain assessment using the visual analog scale (VAS), inflammatory markers from postoperative blood tests, the use of physical restraints, and the administration of antipsychotic medications.

Statistical analysis

Patient data were extracted from electronic medical records for individuals who underwent gastrointestinal surgery and were categorized into two groups: frail and non-frail. Frailty was assessed using the CFS, developed by Rockwood et al. in Canada in 2005 [4]. Patients with a CFS score of 3 or lower were classified as non-frail, while those with a score of 4 or higher, including pre-frail individuals, were classified as frail. Comparative statistical analyses were then conducted between the two groups. Categorical variables were analyzed using Fisher’s exact test. Continuous variables were presented as means and standard deviations, and comparisons were made using the t-test for univariate analysis. For continuous variables that were significantly different in univariate analysis, ROC curves were drawn, and cutoff values were calculated. Binary variables for the continuous variables were converted from the calculated cutoff values, and univariate analysis was performed. Univariate analysis was performed using logistic regression analysis to examine the association between preoperative frailty and non-frailty and perioperative factors, and statistical analysis was performed using EZR ver 1.66. The significance level was set at less than 5%.

## Results

Of the analyzed cohort, 228 patients (70.5%) were classified as non-frail, while 95 patients (29.4%) were identified as frail. The distribution of the CFS scores among the subjects was as follows: CFS 1 - 1.5%; CFS 2 - 14.5%; CFS 3 - 54.4%; CFS 4 - 18.8%; and CFS 5 or higher - 10.8%. Preoperative, intraoperative, and postoperative assessments were conducted between the non-frail and frail groups.

Comparison of preoperative factors between the frail and non-frail groups

Significant differences were observed between the two groups in terms of age (p=0.001), ASA-PS (p<0.001), height (p=0.001), body weight (p=0.005), medication history (p=0.022), smoking history (p<0.001), total protein (p=0.005), albumin (p<0.001), hemoglobin (p=0.01), and hematocrit (p<0.001), all of which were notably lower in the frail group (Table 1).

**Table 1:** Comparison of two groups of non-frailty and frailty preoperative endpoints ASA-PS: American Society of Anesthesiologists - Physical Status; BMI: Body Mass Index; eGFR: Epidermal Growth Factor Receptor; CRP: C-Reactive Protein; TP: Total Protein; WBC: White Blood Cells; RBC: Red Blood Cells; DST: Delirium Screening Tool

Variables		Non-frailty group n=228	Frailty group n=95	Type of values	Statistical Analysis	t-value	p-value
								Age		76.59 (4.88)	80.11 (6.17)	Average (Standard deviation)	t-test	-5.45	<0.001
Gender(%)	Male	141 (61.8)	48 (50.5)	n (%)	Fisher's exact test		0.064
	Female	87 (38.2)	47 (49.5)	n (%)	-		
ASA-PS(%)	1	15 (6.6)	0 (0.0)	n (%)	Fisher's exact test		<0.001
	2	187 (82.0)	62 (65.3)	n (%)	-		
	3	26 (11.4)	33 (34.7)	n (%)	-		
Height (m)		1.59 (0.10)	1.55 (0.11)	Average (Standard deviation)	t-test	3.46	0.001
Weight (kg)		57.98 (12.18)	53.81 (11.65)	Average (Standard deviation)	t-test	2.89	0.005
BMI (kg/m^2^		22.82 (3.90)	22.34 (3.69)	Average (Standard deviation)	t-test	1.03	0.303
Medication history (%)	Unavailable	33 (14.5)	5 (5.3)	n (%)	Fisher's exact test		0.022
	Available	195 (85.5)	90 (94.7)	n (%)	-		
Smoking history (%)	Unavailable	88 (38.6)	58 (61.1)	n (%)	Fisher's exact test		<0.001
	Available	140 (61.4)	37 (38.9)	n (%)	-		
Anamnesis (%)	Unavailable	10 (4.4)	2 (2.1)	n (%)	Fisher's exact test		0.52
	Available	218 (95.6)	93 (97.9)	n (%)	-		
CRP (mg/dL)		0.91 (2.93)	0.84 (2.18)	Average (Standard deviation)	t-test	0.21	0.833
TP (g/dL)		7.04 (0.66)	6.81 (0.69)	Average (Standard deviation)	t-test	2.81	0.005
Albumin (g/dL)		3.79 (0.52)	3.51 (0.53)	Average (Standard deviation)	t-test	4.37	<0.001
Urea (mg/dL)		17.31 (8.04)	19.03 (9.39)	Average (Standard deviation)	t-test	-1.66	0.097
Creatinine (mg/dL)		1.11 (2.23)	1.22 (1.62)	Average (Standard deviation)	t-test	-0.49	0.661
eGFR (mL/min/1.73 ㎡)		61.86 (18.70)	57.70 (20.90)	Average (Standard deviation)	t-test	1.76	0.079
WBC (×10³/μL)		5.92 (2.15)	6.17 (2.36)	Average (Standard deviation)	t-test	1.76	0.358
RBC (×10⁶/μL)		4.11 (0.57)	3.97 (0.86)	Average (Standard deviation)	t-test	1.71	0.087
Hemoglobin (g/dL)		12.33 (1.80)	11.65 (2.89)	Average(Standard deviation)	t-test	2.59	0.01
Hematocrit (%)		37.37 (4.95)	35.12 (5.34)	Average (Standard deviation)	t-test	3.63	<0.001
Platelets (×10³/μL)		238.47 (73.36)	243.73 (83.60)	Average (Standard deviation)	t-test	-0.56	0.574
Preoperative DST	Negative	227 (99.6)	93 (97.9)	n (%)	Fisher's exact test		0.208
	Positive	1 (0.4)	2 (2.1)	n (%)	-		
Preoperative complications: hypertension	Unavailable	93 (40.8)	28 (29.5)	n (%)	Fisher's exact test		0.059
	Available	135 (59.2)	67 (70.5)	n (%)	-		
Preoperative complications: diabetes mellitus	Unavailable	177 (77.6)	63 (66.3)	n (%)	Fisher's exact test		0.037
	Available	51 (22.4)	32 (33.7)	n (%)	-		
Preoperative complications: ischemic heart disease	Unavailable	207 (90.8)	78 (82.1)	n (%)	Fisher's exact test		0.036
	Available	21 (9.2)	17 (17.9)	n (%)	-		
Preoperative complications: cerebrovascular disease	Unavailable	204 (89.5)	73 (76.8)	n (%)	Fisher's exact test		0.005
	Available	24 (10.5)	22 (23.2)	n (%)	-		
Preoperative complications dementia	Unavailable	224 (98.2)	81 (85.3)	n (%)	Fisher's exact test		<0.001
	Available	4 (1.8)	14 (14.7)	n (%)	-		
Psychiatric disorders and alcohol dependence	Unavailable	224 (98.2)	93 (97.9)	n (%)	Fisher's exact test		0.999
	Available	4 (1.8)	2 (2.1)	n (%)	-		

Comparison of intraoperative factors between the frail and non-frail groups

The frail group demonstrated significantly lower intraoperative usage of sevoflurane (p=0.024) and propofol (p<0.001). No significant differences were found in anesthesia duration or opioid administration between the groups (Table 2).

**Table 2:** Comparison of non-frailty and frailty in two groups: intraoperative endpoints

Variables		Non-frailty group n=228	Frailty group n=95	Type of values	Statistical Analysis	t-value	p-value
								Surgery	Scheduled surgery	219 (96.1)	92 (96.8)	n (%)	Fisher's exact test		0.999
Emergency surgery	9 (3.9)	3 (3.2)	n (%)	-											
Duration of anesthesia (minutes)		383.34 (173.53)	354.16 (134.14)	Average (Standard deviation)	t-test	1.43	0.144
Duration of surgery time (minutes)		300.89 (158.28)	274.85 (123.76)	Average (Standard deviation)	t-test	1.46	0.153
Epidural catheter	Unavailable	59 (25.9)	34 (35.8)	n (%)	Fisher's exact test		0.08
	Available	169 (74.1)	61 (64.2)	n (%)	-		
Sevoflurane (%)		163.36 (131.98)	131.36 (60.51)	Average (Standard deviation)	t-test	2.26	0.024
Propofol (mg)		89.99 (35.31)	75.12 (28.46)	Average (Standard deviation)	t-test	3.36	<0.001
Fentanyl (μg)		192.22 (142.08)	208.63 (161.24)	Average (Standard deviation)	t-test	0.81	0.365
Remifentanil (mg)		2.13 (1.58)	1.98 (1.33)	Average (Standard deviation)	t-test	0.81	0.42
Estimated blood loss(ml)		69.90 (177.33)	63.82 (141.78)	Average (Standard deviation)	t-test	0.29	0.767
Blood transfusion	Unavailable	217 (95.6)	87 (92.6)	n (%)	Fisher's exact test		0.28
	Available	10 (4.4)	7 (7.4)	n (%)	-		

Comparison of postoperative factors between the frail and non-frail groups

Postoperative variables showing significant reductions in the frail group included length of hospital stay (p=0.002), VAS pain score on postoperative day two (p=0.025), albumin (p=0.04), white blood cell count (p=0.042), hemoglobin (p=0.023), hematocrit (p=0.032), incidence of physical restraint (p=0.024), and postoperative DST scores (p=0.02) (Table 3).

**Table 3:** Comparison of non-frailty and frailty in two groups: postoperative endpoints POD: Postoperative Day; VAS: Visual Analogue Scale, ranging from 0 to 100 (0=no pain, 100=worst imaginable pain); CRP: C-Reactive Protein; WBC: White Blood Cells; DST: Delirium Screening Tool

Variables		Non-frailty group, n=228	Frailty group, n=95	Type of values	Statistical analysis	t-value	p-value
								Length of hospital stay (day)		17.90 (12.80)	24.14 (22.58)	Average (Standard deviation)	t-test	-3.13	0.002
Day of getting out of bed (day)		1.19 (2.09)	1.12 (0.41)	Average (Standard deviation)	t-test	0.35	0.721
Walking start date (day)		1.46 (2.21)	1.53 (1.34)	Average (Standard deviation)	t-test	-0.25	0.801
Postoperative Pain Day 1 VAS (mm)		34.54 (22.81)	35.11 (26.04)	Average (Standard deviation)	t-test	-0.19	0.846
Postoperative Pain Day 2 VAS (mm)		26.36 (20.74)	32.11 (21.33)	Average (Standard deviation)	t-test	-2.24	0.025
CRP (mg/dL)		4.46 (3.77)	4.50 (2.99)	Average (Standard deviation)	t-test	0.21	0.916
WBC (×10³/μL)		9.35 (3.05)	10.11 (3.00)	Average (Standard deviation)	t-test	-2.04	0.042
Platelets (×10³/μL)		208.06 (64.30)	212.52 (73.67)	Average (Standard deviation)	t-test	-0.53	0.587
Use of physical restraints	Unavailable	223 (97.8)	87 (91.6)	n (%)	Fisher's exact test		0.024
	Available	5 ( 2.2)	8 ( 8.4)	n (%)	-		
The administration of antipsychotic medications	Unavailable	169 (74.1)	68 (71.6)	n (%)	Fisher's exact test		0.679
	Available	59 (25.9)	27 (28.4)	n (%)	-		
Postoperative DST	Negative	216 (94.7)	82 (86.3)	n (%)	Fisher's exact test		0.02
	Positive	12 ( 5.3)	13 (13.7)	n (%)	-		

Perioperative predictive factors and cutoff value determination

**Figure 2:**
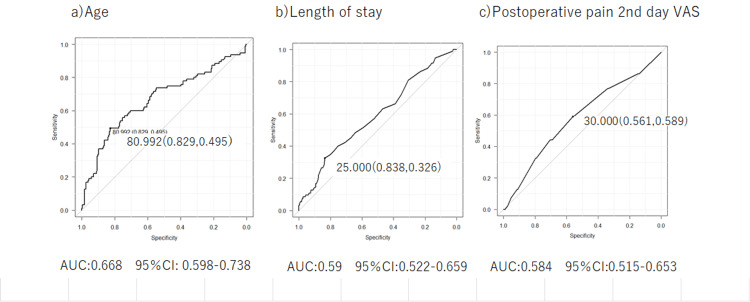
ROC curves of age, length of hospital stay, and postoperative pain for discriminating between frail and non-frail patients VAS: Visual Analog Scale

From preoperative to postoperative, ROC curves were drawn, and cutoff values were calculated for three items that showed significant differences: age, length of hospital stay, and postoperative pain on day two (Figure 2). The calculated cutoff values were as follows: age - 80.9 years (sensitivity: 49.5%, specificity: 82.9%); hospital stay - 25 days (sensitivity: 32.6%, specificity: 83.8%); and postoperative day two VAS score - 30 (sensitivity: 58.9%, specificity: 56.1%).

Logistic regression analysis using frailty status as the dependent variable

To identify perioperative factors associated with frailty, both univariate and multivariate logistic regression analyses were performed using variables that demonstrated significant differences: age, length of hospital stay, presence of physical restraint, postoperative DST score, and VAS pain score on postoperative day two (Table 4).

**Table 4:** Results of logistic regression analysis using frailty as the objective variable ASA-PS: American Society of Anesthesiologists - Physical Status; DST: Delirium Screening Tool; POD: Postoperative Day; VAS: Visual Analog Scale

Variables	Univariate analysis			Multivariate analysis		
	Odds ratio	95%CI	p-value	Odds ratio	95%CI	p-value
Age	3.77	2.280-6.240	<0.001	3.97	2.26000-6.9900	<0.001
Number of days in hospital	2.5	1.440-4.360	<0.001	1.9	1.01000-3.5800	0.048
ASA-PS	4.3	2.46000-7.5000	<0.001	4.27	2.31000-7.9000	<0.001
Presence or absence of physical restraint	4.1	1.310-12.9	0.0157	1.45	0.33000-6.3500	0.623
Postoperative DST	2.85	1.250-6.51	<0.001	1.17	0.40600-3.3800	0.770
Postoperative Pain Day 2 VAS	1.84	1.130-2.990	0.014	1.71	0.99300-2.9600	0.052

In univariate analysis, all six variables were significantly associated with frailty: age (odds ratio (OR): 3.77, 95% confidence interval (CI): 2.280-6.240; p=<0.001), hospital stay (OR: 2.5, 95% CI: 1.440-4.360; p=<0.001), ASA-PS (OR: 4.3, 95% CI: 2.460-7.500; p=<0.001), presence of physical restraint (OR: 4.1, 95% CI: 1.310-12.9; p=0.0157), postoperative DST (OR: 2.85, 95% CI: 1.250-6.51; p=<0.001), and postoperative day two VAS score (OR: 1.84, 95% CI: 1.130-2.990; p=0.014). Subsequent multivariate analysis revealed the following adjusted odds ratios: age (OR: 3.97, 95% CI: 2.260-6.990; p=<0.001), hospital stay (OR: 1.9, 95% CI: 1.010-3.580; p=0.048), ASA-PS (OR: 4.27, 95% CI: 2.310-7.900; p=<0.001), presence of physical restraint (OR: 1.45, 95% CI: 0.330-6.350; p=0.623), postoperative DST (OR: 1.17, 95% CI: 0.406-3.380; p=0.770), and postoperative day two VAS score (OR: 1.71, 95% CI: 0.993-2.960; p=0.052).

## Discussion

According to a systematic review examining postoperative outcomes in frail older adults, frailty has been associated with adverse surgical outcomes, including increased 30-day, 90-day, and one-year mortality rates, postoperative complications, and prolonged hospital stays [8]. These findings underscore the importance of preoperative frailty assessment as a critical indicator of healthcare quality in an aging society such as Japan. The prevalence of frailty among surgical patients in the present study was 29.4%, which falls within the range reported by previous studies focusing on surgical patients [9,10]. Prior studies have reported that, among patients undergoing gastrointestinal surgery - particularly elderly patients with colorectal cancer - preoperative frailty is significantly associated with increased risks of postoperative complications, extended hospitalization, and discharge to rehabilitation or long-term care facilities rather than to home [11,12]. Although flail was not observed to be associated with postoperative complications in this study, it may have been influenced by the different types of surgeries (upper gastrointestinal, hepatobiliary, and lower gastrointestinal), which were performed in different degrees of invasiveness.

Preoperative frailty and postoperative pain

This study investigated the impact of preoperative frailty on postoperative pain. Logistic regression analysis showed that frail patients tended to have a 1.71-fold increased risk of postoperative pain, which is consistent with findings from previous studies. Elderly patients, particularly those with frailty, face challenges in pain assessment due to reduced physiological reserves, multiple comorbidities, and cognitive impairments, often leading to undertreatment of pain [13]. Furthermore, reports indicate that frail patients tend to consume nearly all prescribed opioids, suggesting the complexity of pain management in this population [14]. Given this background, no prior studies have specifically used the VAS to assess postoperative pain in frail patients undergoing gastrointestinal laparoscopic surgery. One of the most important findings of this study is that a cutoff value of 30 was calculated for the VAS score on postoperative day two. A VAS score exceeding 30 mm has been reported as indicating pain that "interferes with daily life" [15]. We believe that this cutoff value of 30 is a clinically crucial indicator for the timely implementation of appropriate intervention and treatment. The lower tolerance or coping ability for postoperative pain in frail patients may also be associated with this result. Regarding the significant difference in VAS from the second day of postoperative pain, it is possible that the pain is exacerbated with increased activity as patients are weaned from bed, and that frail patients have a low tolerance or low coping ability for postoperative pain. In addition, one study found that patients with hearing loss received predominantly lower doses of analgesics than patients with other risk factors; when using Patient Controlled Analgesia (“PCA”) pumps, the ability of older patients to manage the pump should be reevaluated frequently. Pain is a factor in inducing delirium [1].

Preoperative frailty and postoperative DST

The incidence of postoperative delirium generally ranges from 5% to 15% in general surgical wards and rises to approximately 30% in patients undergoing cardiac surgery via thoracotomy. In a previous study conducted at our institution, the overall DST positivity rate was 6.4%, with no significant gender differences. According to Arita et al., 13.3% of patients aged 70 and older undergoing colorectal cancer surgery were diagnosed with postoperative delirium, with identified risk factors including impaired preoperative physical function, reduced lower limb muscle strength, malnutrition, and routine benzodiazepine use [16]. In the present study, the overall DST positivity rate was 7.7%, and among frail patients, it was 13.6%. Although our cohort included a variety of procedures - upper gastrointestinal, hepatobiliary-pancreatic, and lower gastrointestinal surgeries - the incidence of delirium among frail patients closely mirrored that reported in prior studies focused specifically on colorectal surgery. Logistic regression analysis suggested a potential association between frailty and DST positivity (OR: 1.17, 95% CI: 0.406-3.380; p=0.01). However, as the confidence interval included 1, the effect could not be deemed statistically significant. Nonetheless, the trend is consistent with earlier findings, reinforcing the need for individualized risk assessment based on surgical invasiveness and the operative site. Additionally, while not diagnostic for delirium, 69 of 298 DST-negative patients (23.1%) had been administered haloperidol. A study by Kalisvaart et al. reported that prophylactic use of haloperidol did not reduce the incidence of delirium but did mitigate its severity and shorten its duration [17]. In the present study, the use of haloperidol prior to formal delirium diagnosis may have contributed to the relatively low DST positivity rate of 7.7%.

Preoperative frailty and ASA-PS

The ASA-PS serves as a widely utilized tool that allows clinicians across various specialties to rapidly assess a patient's overall physical health status [18]. Applicable to both elective and emergency cases across all age groups, ASA-PS has been shown to correlate with postoperative delirium, mortality, and complications, particularly in older adults undergoing diverse surgical procedures [19]. In the present study, logistic regression analysis supported the potential influence of ASA-PS on frailty status (OR: 4.27, 95% CI: 2.310-7.900; p=0.01), as well as a possible association between frailty and postoperative DST positivity (OR: 1.17, 95% CI: 0.406-3.380; p=0.01), though the latter did not reach statistical significance due to the confidence interval encompassing 1. Kim et al. reported that a multidimensional frailty score derived from Comprehensive Geriatric Assessment (CGA) outperformed ASA-PS in predicting postoperative mortality and length of hospital stay in elderly surgical patients (AUROC: 0.821 vs. 0.647; p=0.01) [20]. In populations undergoing abdominal surgery, the ASA-PS has been associated with poor prognostic outcomes. Among the predictive scales examined, only the CFS was shown to significantly enhance the predictive value of the ASA-PS [3]. Furthermore, the use of three preoperative risk stratification tools - ASA-PS, the Charlson Comorbidity Index (CCI), and the Rockwood Frailty Index (FI) - has been linked to an increased risk of postoperative pneumonia [21]. Based on these findings and our study results, the observed association between frailty and ASA-PS is consistent with prior research. A combined assessment using both the CFS and ASA-PS may offer a more comprehensive preoperative evaluation strategy, potentially contributing to the reduction of postoperative complications and mortality in elderly surgical patients.

Limitations of the study

This study has several limitations. First, it is a retrospective study, and the assessment of frailty was based on information obtained from past medical records, which may be somewhat inaccurate. Second, the study population included a heterogeneous group of gastrointestinal surgical procedures - encompassing upper gastrointestinal, hepatobiliary-pancreatic, and lower gastrointestinal surgeries. This diversity likely introduced variability in factors such as operative time, postoperative inflammatory response, timing of mobilization, and pain management strategies. Postoperative management protocols also differed by surgical type. In terms of pain control, some patients did not receive epidural catheterization due to anticoagulant therapy, while others had their PCA infusion rates adjusted or discontinued to facilitate early ambulation. These variations suggest that further analysis considering the background of pain management strategies would have been valuable. Additionally, it remains unclear whether frail patients were physically capable of self-administering PCA analgesia, whether patients with hearing impairment received appropriate pain management, and whether pain was adequately assessed - indicating the need for further investigation. Although this study successfully identified the rate of DST positivity as a proxy for postoperative delirium, 23.1% of DST-negative patients were found to have received haloperidol postoperatively. This raises questions regarding the accuracy of DST assessments and underscores the need to examine the clinical rationale behind haloperidol administration to clarify whether subclinical or misclassified delirium may have occurred.

## Conclusions

Intraoperative and postoperative factors associated with frail patients undergoing gastrointestinal laparoscopic surgery included age, length of hospital stay, ASA-PS, presence of physical restraint, positive postoperative DST rate, and postoperative pain. Hospital length of stay, physical restraint, and pain were all associated with the risk of delirium, suggesting that frail patients are more prone to delirium from these factors. Appropriate pain management, regular frailty screening utilizing both the CFS and ASA-PS classification, and interventions for delirium prevention may reduce the risk of perioperative complications.

## References

[1] Inouye SK, Westendorp RG, Saczynski JS (2014). Delirium in elderly people. Lancet.

[2] Dent E, Kowal P, Hoogendijk EO (2016). Frailty measurement in research and clinical practice: a review. Eur J Intern Med.

[3] Satake S, Arai H (2020). The revised Japanese version of the cardiovascular health study criteria (revised J-CHS criteria). Geriatr Gerontol Int.

[4] Rockwood K, Song X, MacKnight C, Bergman H, Hogan DB, McDowell I, Mitnitski A (2005). A global clinical measure of fitness and frailty in elderly people. CMAJ.

[5] Lamberink K, Vermeeren YM, Moes AD, Mulderij J, Rootjes PA, Zomer TP (2024). Usefulness of the clinical frailty scale in patients with end-stage kidney disease. Clin Kidney J.

[6] Miller D, Lewis SR, Pritchard MW, Schofield-Robinson OJ, Shelton CL, Alderson P, Smith AF (2018). Intravenous versus inhalational maintenance of anaesthesia for postoperative cognitive outcomes in elderly people undergoing non-cardiac surgery. Cochrane Database Syst Rev.

[7] Kubota K, Suzuki A, Ohde S, Yamada U, Fujitani I, Koitabashi A (2020). Development of a simple and practical delirium screening tool for use in surgical wards. J Nurs Res.

[8] Lin HS, Watts JN, Peel NM, Hubbard RE (2016). Frailty and post-operative outcomes in older surgical patients: a systematic review. BMC Geriatr.

[9] Hewitt J, Long S, Carter B, Bach S, McCarthy K, Clegg A (2018). The prevalence of frailty and its association with clinical outcomes in general surgery: a systematic review and meta-analysis. Age Ageing.

[10] McGovern J, Dolan RD, Horgan PG, Laird BJ, McMillan DC (2022). The prevalence and prognostic value of frailty screening measures in patients undergoing surgery for colorectal cancer: observations from a systematic review. BMC Geriatr.

[11] Makary MA, Segev DL, Pronovost PJ (2010). Frailty as a predictor of surgical outcomes in older patients. J Am Coll Surg.

[12] Ommundsen N, Wyller TB, Nesbakken A, Bakka AO, Jordhøy MS, Skovlund E, Rostoft S (2018). Preoperative geriatric assessment and tailored interventions in frail older patients with colorectal cancer: a randomized controlled trial. Colorectal Dis.

[13] Van Zundert TC, Gatt SP, van Zundert AA (2023). Anesthesia and perioperative pain relief in the frail elderly patient. Saudi J Anaesth.

[14] Auckley ED, Bentov N, Zelber-Sagi S, Jeong L, Reed MJ, Bentov I (2021). Frailty status as a potential factor in increased postoperative opioid use in older adults. BMC Geriatr.

[15] Ahlers SJ, van Gulik L, van der Veen AM (2008). Comparison of different pain scoring systems in critically ill patients in a general ICU. Crit Care.

[16] Arita A, Takahashi H, Ogino T (2021). Grip strength as a predictor of postoperative delirium in patients with colorectal cancers. Ann Gastroenterol Surg.

[17] Kalisvaart KJ, de Jonghe JF, Bogaards MJ (2005). Haloperidol prophylaxis for elderly hip-surgery patients at risk for delirium: a randomized placebo-controlled study. J Am Geriatr Soc.

[18] Ferrari L, Leahy I, Staffa SJ, Berry JG (2021). The pediatric-specific American Society of Anesthesiologists physical status score: a multicenter study. Anesth Analg.

[19] Aitken R, Harun NS, Maier AB (2021). Which preoperative screening tool should be applied to older patients undergoing elective surgery to predict short-term postoperative outcomes? Lessons from systematic reviews, meta-analyses and guidelines. Intern Emerg Med.

[20] Kim SW, Han HS, Jung HW, Kim KI, Hwang DW, Kang SB, Kim CH (2014). Multidimensional frailty score for the prediction of postoperative mortality risk. JAMA Surg.

[21] Laporta ML, Kruthiventi SC, Mantilla CB (2021). Three risk stratification tools and postoperative pneumonia after noncardiothoracic surgery. Am Surg.

